# Supplementary material for: Bacterial Lipid II Analogs: Novel In Vitro Substrates for Mammalian Oligosaccharyl Diphosphodolichol Diphosphatase (DLODP) Activities
Source: Molecules. 2019 Jun 6;24(11):2135. doi: 10.3390/molecules24112135 (PMC6600155; doi:10.3390/molecules24112135)
Supplement: Supplementary file 1 [file molecules-24-02135-s001.pdf]

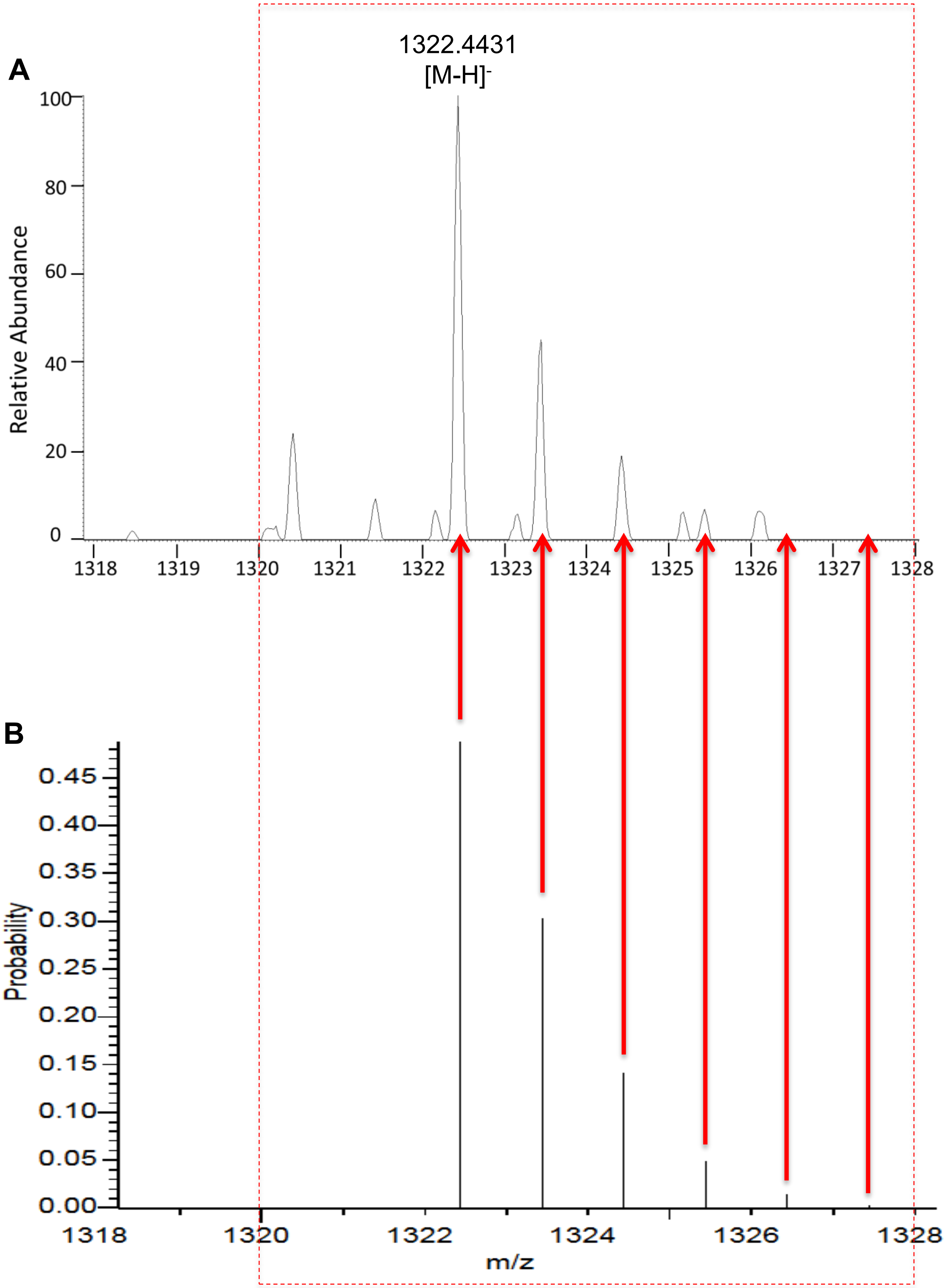

The observed isotopolog cluster centered around the 1322.4431 [M-H]- signal (A) is aligned with the theoretical cluster (B) computed for GM5P (22).
